# Effect of kinesiophobia on postoperative rehabilitation outcomes in patients with cervical spondylotic myelopathy: a cross-sectional study

**DOI:** 10.1186/s13018-024-04819-4

**Published:** 2024-08-09

**Authors:** Yaqiong Chen, Guiqin Zhong, Shichai Zhong, Jingjing Lin, Yanjuan Lin

**Affiliations:** 1 Department of Nursing, Union Hospital, Fujian Medical University, Xinquan Road 29, Fuzhou, Fujian 350001 China; 2 Department of Cardiovascular Surgery, Union Hospital, Fujian Medical University, Fuzhou, Fujian China; 3 Department of Neurosurgery, Union Hospital, Fujian Medical University, Fuzhou, Fujian China

**Keywords:** Cervical spondylotic myelopathy, Exercise fear, TSK score, Cervical spine function

## Abstract

**Objective:**

This study aims to investigate the occurrence of postoperative kinesiophobia in patients with CSM and compare the postoperative recovery of patients with and without kinesiophobia to understand its influence on clinical outcomes in CSM.

**Methods:**

Between November 2020 and November 2022, surgical treatment was performed in the neurosurgical wards of 2 Grade III Class A general public hospitals in the Fujian Province. The demographic and disease data of the patients were collected, and patients were divided into a kinesiophobia group and non-kinesiophobia group according to the Tampa kinesiophobia Scale (TSK). The cervical dysfunction index, cervical Japanese Orthopaedic Association (JOA) rating, self-anxiety rating, and activity of daily living rating scales were collected three months postoperatively. The influence of postoperative kinesiophobia on early rehabilitation was also analysed.

**Results:**

A total of 122 patients were an average age of (55.2 ± 10.3) years included in this study. The average score of kinesophobia after surgery was 41.2 ± 4.5, with an incidence of 75.4%. Multivariate logistic regression analysis showed that age (OR = 1.105, 95% CI = 1.014–1.204), neck disability index (NDI) (OR = 1.268, 95% CI = 1.108–1.451), diabetes mellitus (OR = 0.026, 95% CI = 0.001–0.477), and Japanese Orthopaedic Association (JOA) score (OR = 0.698, 95% CI = 0.526–0.927) were associated with the occurren.

**Conclusion:**

Doctors should be aware of kinesiophobia in patients with CSM. Education regarding kinesiophobia, strategies to avoid it, and treatment strategies using a multidisciplinary approach can improve recovery outcomes.

## Introduction

Cervical spondylotic myelopathy (CSM) accounts for 10–15% of all cervical spondylotic diseases and is the most common cause of spinal cord dysfunction in adults worldwide. It is one of the more serious forms of cervical spondylosis [1]. Without timely surgery to relieve spinal cord compression and restore the normal curvature of the cervical spine, this disease can lead to progressive disability and affect quality of life. Characteristic symptoms of CSM include loss of hand flexibility, muscle weakness, limb stiffness, urinary urgency, frequent urination or hesitation, limb spasms, and gait disorders [2, 3]. In North America, the incidence of hospitalizations due to cervical myelopathy is approximately 404/100,000 person-years [4], and the incidence of surgically treated patients with cervical myelopathy is at least 1.6 per 100,000 residents-year [5]. Postoperative exercise is a crucial part of patients’ rehabilitation; however, many patients experience a reduction in movement for a variety of reasons, namely, fear of exercising [6]. Therefore, we speculate that fear of exercising may affect patients’ participation in postoperative rehabilitation exercises, which may in turn influence the effectiveness of rehabilitation, although the objective improvements in sensory, motor, and bladder functions are expected to occur regardless of exercise.

Kinesiophobia, is a psychological phenomenon characterized by the fear that daily activities or physical exercises will cause injury or re-injury [7]. Fear avoidance behavior has been well studied in patients with diseases such as spinal osteoarthritis. In a study of spinal degenerative diseases, kinesiophobia could significantly predict pain, disability, and physical health of patients 6 months later [8]. Moreover, a national multicenter cohort observational study demonstrated that kinesiophobia is common in those with neck and low back pain; nearly 80% of patients had a clear kinesiophobia score [9, 10]. Previous studies have also confirmed that kinesiophobia and catastrophic behaviors can lead to recurrent neck pain or changes in the somatosensory system [11], which may alter the afferent input to the higher centers of the neck and impair neck proprioception.

There are few studies on the correlation between postoperative kinesiophobia and rehabilitation outcomes in CSM patients. Therefore, this study aimed to investigate the occurrence of postoperative kinesiophobia in patients with CSM and compare the postoperative recovery of patients with and without kinesiophobia to understand the influence of kinesiophobia on the postoperative clinical outcomes in CSM.

## Materials and methods

### Ethical considerations

This study was registered with the Chinese Clinical Trials Registry (ChiCTR2000040508) and conducted in accordance with the Declaration of Helsinki. It was approved by the Ethics Committee of our institution (Ethics number: 2020YF034-01), and all volunteers provided written consent.

**Fig. 1:**
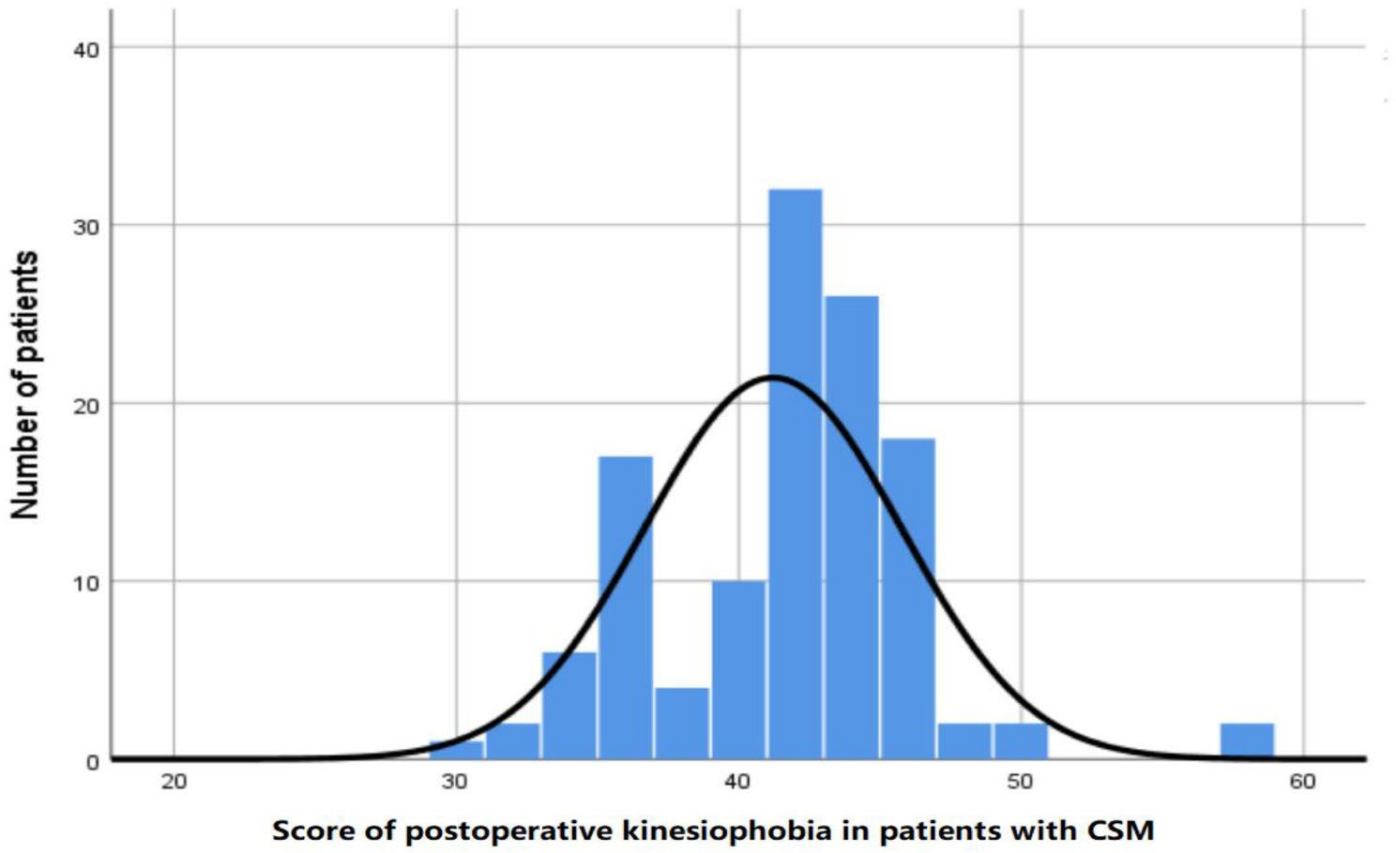
Sum scores of TSK

All eligible follow-up patients were included in the study without calculating a sample size. A total of 150 patients with CSM were invited to participate (Fig. 1). The study was conducted in the neurosurgery wards of two Grade-III Class A general public hospitals in Fujian Province between November 2020 and November 2022. The inclusion criteria were as follows: (1) age ≥ 18 years; (2) Preoperative confirmation of cervical disc herniation and compression by MRI and CT, accompanied by typical physical signs of cervical spondylosis, leading to a diagnosis of cervical spondylotic myelopathy. Typical physical signs include neck pain, stiffness, shoulder pain, arm numbness, finger numbness, and headaches, as well as neurological deficits such as muscle weakness, spasticity, and gait disturbances indicative of spinal cord dysfunction; (3) no absolute contraindications to surgical treatment; (4) informed consent; (5) no communication difficulties. Exclusion criteria included: (1) presence of other pain-causing diseases; (2) history of cervical spine surgery, cerebrovascular disease, or neurological disease; (3) long-term confinement to bed before surgery, loss of the ability to walk, or inability to self-care; (4) postoperative complications or adverse reactions leading to limited activities; (5) postoperative neurological dysfunction.

Following general anesthesia, the patient was placed in a supine position. A neuroelectrophysiological (neuro-navigation system) probe was connected to the instrument, and routine disinfection of the surgical filed and draping. A transverse incision was made on the right/left anterior neck striae. The skin, subcutaneous tissue, and platysma muscle were incised. The subcutaneous tissue on the right anterior neck was bluntly separated along the lower part of the platysma muscle, and the fascia was bluntly separated longitudinally with scissors inside the sternocleidomastoid muscle, exposing the space between the internal carotid artery sheath and tracheoesophagus. The hyperplastic bone was removed using an ultrasonic bone knife under the microscope, and the spinal canal was fully decompressed. Finally, the cervical curvature was improved through titanium alloy screw fixation.

### Data collection

Trained researchers communicated with eligible and consenting patients before the study and completed general information questionnaires after admission. The questionnaires included items such as age, sex, marital status, education, common underlying conditions, previous cervical spondylosis surgery, smoking, and alcohol consumption. The kinesiophobia questionnaire was completed within 24 h after recovery from anesthesia, when the patient’s condition was stable. Three months after surgery, the researchers contacted the patients via telephone and arranged for a follow-up at our hospital in the following days to complete the Grip strength test, Neck Disability Index (NDI) score, Cervical Spine Japanese Orthopaedic Association (JOA) score, Self-rating Anxiety Scale (SAS), and Functional State Assessment of Activities of Daily Living (ADL) score. If the patient was illiterate, the investigator helped in filling out the form by asking questions and noting answers, without giving any suggestive guidance. The questionnaire was collected on the spot and checked carefully. If there was any missing item, the patient was reminded to complete it. Patient data were collected and collated by two researchers against the electronic medical records.

### Research tools

#### General data questionnaire

A general data questionnaire was developed based on a literature search and expert consultation, covering demographic data, disease history, and hospitalization-related medical information.

#### Tampa scale of kinesiophobia (TSK)

The 17-item TSK was utilized to assess kinesiophobia (e.g.,“Simply being careful that I do not make any unnecessary movements is the safest thing I can do to prevent my pain from worsening”) and fear of (re)injury (e.g., “Pain always means I have injured my body”), with scores ranging from 17 to 68 [12]. Participants are asked to rate each item, 4 being negatively worded and reversescored (items 4, 8, 12, and 16), on a 4-point Likert scale with scoring alternatives ranging from ‘‘strongly disagree’’ to ‘‘strongly agree.’’ Higher scores indicate a greater fear of exercise (a score of ≥ 37 indicates kinesiophobia) [13]. The TSK has demonstrated good reliability, with a Cronbach’s α > 0.7 and retest reliability of Pearson’s *r* > 0.7.

#### Self-rating anxiety scale (SAS)

Developed by Professor Zung [14], the SAS comprises 20 items graded on a Likert-4 scale from “not at all” to “almost always”. Scores on items 5, 9, 13, 17, and 19 are reverse-scored. The total score is the sum of all 20 items, with a cutoff of 50 points. Scores of 50–59 indicate mild anxiety, 60–69 indicate moderate anxiety, and > 69 indicate severe anxiety.

#### Muscle strength evaluation

Grip strength, a key indicator of overall muscle strength and related to prognosis and quality of life [15], was assessed using a handheld grip tester (Xiangshan EH101, specification: 19.5 cm x 13 cm x 3 cm). Initial evaluation was performed with the free hand muscle strength test, and if the muscle strength was > grade 3, the grip strength test was administered.

#### Cervical spine JOA score (JOA)

The latest version of the JOA score scale was utilized, consisting of six areas: upper limb motor dysfunction, lower limb motor dysfunction, upper limb sensory function, trunk sensory function, lower limb sensory function, and bladder function. Scores range from 0 to 17, with lower scores indicating more severe dysfunction. A JOA score of 9–13 is classified as moderate, while < 9 is classified as severe [16].

#### Neck disability index (NDI)

The NDI comprises 10 subsections assessing pain intensity, personal care, weightlifting, reading, headache, concentration, work, driving, sleep, and recreation. Scores range from 0 to 50, with higher scores indicating more severe neck dysfunction. The NDI has demonstrated good retest reliability (Pearson’s *r* > 0.80) and internal consistency (Cronbach’s α > 0.70) [17].

#### Functional state assessment of activities of daily living (ADL)

This scale assesses the degree of independence in ADL, with 10 items and a maximum score of 100. Scores of 1–24 indicate total dependence, 25–49 indicate severe dependence, 50–74 indicate moderate dependence, 75–90 indicate mild dependence, 91–99 indicate minimal dependence, and 100 indicates complete independence. The scale has high inter-score reliability (*r* = 0.95) and internal reliability (*r* = 0.89) [18].

### Statistical analysis

Statistical analysis was performed using SPSS 26.0 statistical software (SPSS; Chicago, IL, USA). Descriptive statistics were used to summarize the demographic and clinical characteristics of the study population. Continuous variables were expressed as mean ± standard deviation (SD), and categorical variables were expressed as frequencies and percentages.

Differences between groups were analyzed using the Mann-Whitney U test for continuous variables that did not follow a normal distribution and the chi-squared test for categorical variables. Spearman correlation analysis was used to examine the correlation between kinesiophobia and other continuous variables.

Univariate logistic regression analysis was initially conducted to identify potential predictors of kinesiophobia. Variables with *p* < 0.05 in the univariate analysis were included in the multivariate logistic regression analysis to identify independent predictors of kinesiophobia. Collinearity diagnostics were performed to assess the relationship between variables.

## Results

### Duration and manner of follow-up

The early follow-up data were acquired through hospital examinations, while the late follow-up information was obtained via telephone contact. All patients are followed up postoperatively by two designated researchers. Of the 150 eligible patients, 28 did not participate, resulting in 122 patients completing the three-month follow-up (52 females and 70 males, average age 55.20 ± 10.31 years; see Fig. 1).

### Demographics

**Fig. 2:**
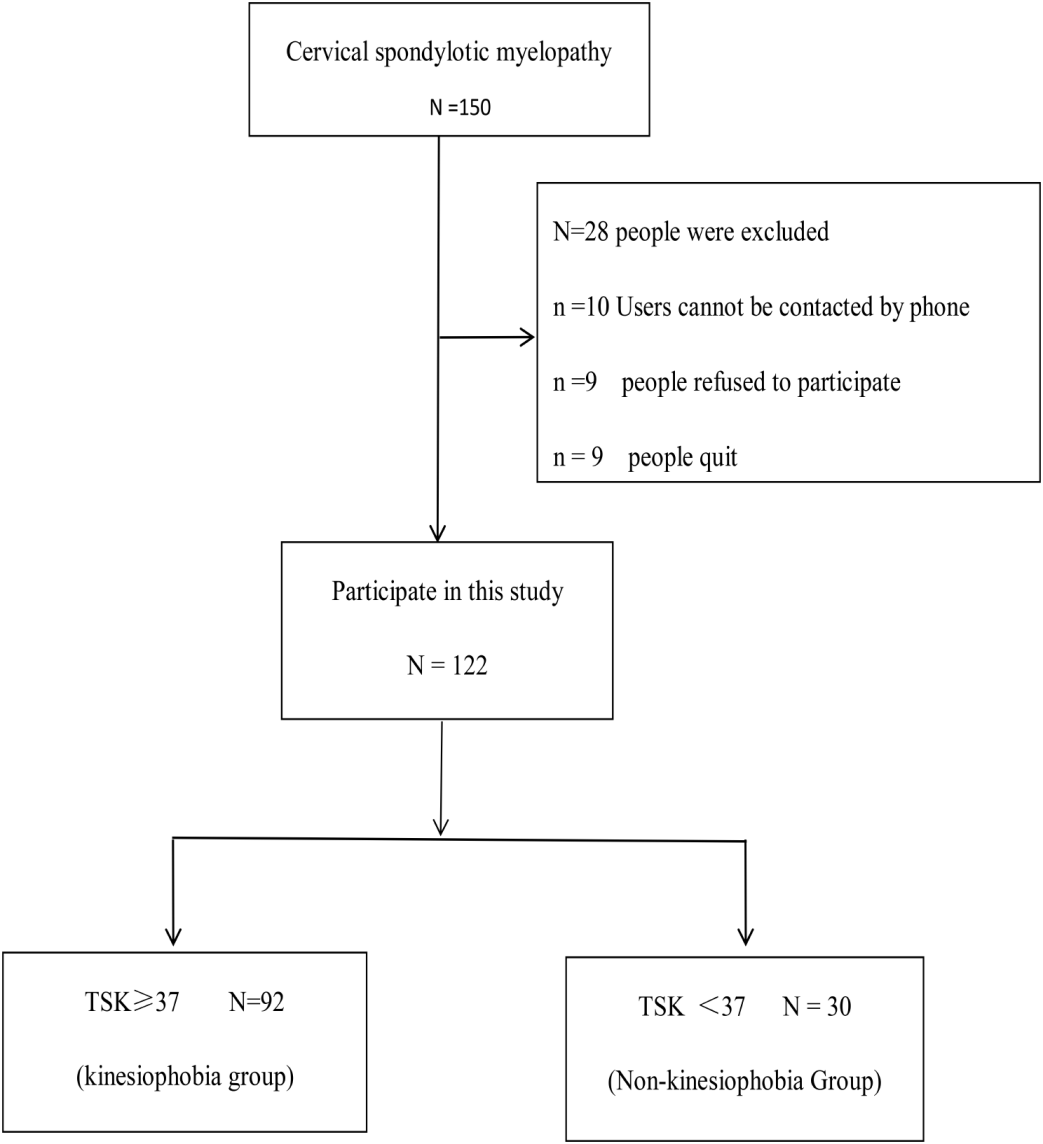
Screening flow chart of patients with cervical spondylotic myelopath

The total Tampa Scale of Kinesiophobia (TSK) scores ranged from 30 to 57, with an average score of 41.2 ± 4.5. The incidence of kinesiophobia after CSM surgery was 75.4% (92/122) (see Fig. 2). The majority of married patients were female (95.08%). Additionally, 14 cases (11.48%) were complicated with diabetes, 24 cases (19.67%) with hypertension, and five had a history of cervical spine surgery. The demographic characteristics and baseline characteristics of the participants are shown in Table 1.

**Table 1:** Preoperative data analysis of patients with and without kinesophobia

Variable	Kinesophobia (*n* = 92)	Nonkinesophobia (*n* = 30)	*P* value
Age (years)	57.4 ± 8.7	48.3 ± 12.0	0.005
Gender (Male), n(%)	54 (58.7)	16 (53.3)	0.606
BMI (kg/m^ˆ2^) (≥ 24), n(%)	37 (40.2)	9 (30.0)	0.316
Marital status (Married), n(%)	86 (93.5)	28 (93.3)	0.978
Education levels (high school or below), n(%)	75 (81.5)	17(56.7)	0.006
Current smoking, n (%)	40 (43.5)	13 (43.3)	0.989
Current drinking, n (%)	6 (6.5)	3 (10.0)	0.527
Diabetes mellitus, n (%)	8 (8.7)	6 (20.0)	0.092
Hypertension, n (%)	16 (17.4)	8 (26.7)	0.267
Previous history of other surgeries, n (%)	52 (56.5)	12 (40.0)	0.116
History of cervical spine sports injury, n (%)	3 (3.3)	2 (6.7)	0.414
Duration of symptoms (months)	24.3 ± 30.9	18.0 ± 24.5	0.021
Time of movement out of bed after surgery (hours)	51.8 ± 28.9	42.93 ± 23.0	0.273
Preoperative occupational status (employment), n (%)	67 (72.8)	23 (76.7)	0.678
Length of stay (hours)	16.1 ± 5.2	16.93 ± 5.3	0.426
Payment method of medical expenses (At one’s own expense), n (%)	30 (32.6)	9 (30.0)	0.790
Grip strength test			
Left hand grip strength test (kg)	19.6 ± 10.3	22.6 ± 10.4	0.908
Right hand grip strength test (kg)	22.0 ± 11.9	27.4 ± 9.7	0.346

Continuous normally distributed variables were expressed as mean (standard deviation) and categorical data are given as the counts and percentage (n, %)

### Univariate analysis of postoperative kinesiophobia in CSM patients

Patients with high levels of kinesiophobia were older, less educated, had a longer disease duration, and a higher incidence of diabetes compared to those with low levels of kinesiophobia. No significant differences were observed between the two groups in terms of gender, marital status, medical payment method, occupational status, length of stay, and grip strength test results three months postoperatively. Additional basic characteristics are detailed in Table 1.

### Correlation analysis between kinesiophobia and various scales in postoperative CSM patients

Table 2 presents the correlation between kinesiophobia scores and Self-rating Anxiety Scale (SAS), Neck Disability Index (NDI), Cervical Spine Japanese Orthopaedic Association (JOA), and Functional State Assessment of Activities of Daily Living (ADL). Kinesiophobia showed a significant negative correlation with the cervical JOA score (*r* = -0.681, *P* < 0.001), a strong positive correlation with the NDI (*r* = 0.715, *P* < 0.001), and a slight positive correlation with the SAS (*r* = 0.282, *P* = 0.002). It also demonstrated a significant negative correlation with early quality of life (*r* = -0.608, *P* < 0.001). Higher levels of exercise fear in patients with CSM after surgery were associated with poorer body function and cervical spine function performance 3 months postoperatively and a higher incidence of anxiety, while lower levels of exercise fear were associated with a higher quality of life.

**Table 2:** Correlation analysis of postoperative kinesophobia and scores of each scale in CSM patients

Variable	Score	*R* value	*P* value
JOA	13.4 ± 3.4	-0.681	0.000
NDI	24.43 ± 12.1	0.715	0.000
SAS	29.8 ± 7.8	0.282	0.002
ADL	89.9 ± 12.8	-0.608	0.000

JOA: Cervical Spine Japanese Orthopaedic Association; NDI: Neck Disability Index; SAS: Self-rating Anxiety Scale; ADL: Assessment of Activities of Daily Living and Functional State. Continuous normally distributed variables were expressed as mean (standard deviation)

### Multiple logistic regression analysis

Using exercise fear as the dependent variable, variables with *P* < 0.1 in the univariate analysis were included (Table 3). Eight variables ultimately met the inclusion criteria: age, education (high school or below), duration of symptoms, diabetes (yes), JOA score, NDI score, SAS score, and ADL score. Original values of the continuous variables were entered, while categorical variables were assigned values. Multivariate logistic regression analysis revealed that age (OR = 1.105, 95% CI = 1.014–1.204), NDI (OR = 1.268, 95% CI = 1.108–1.451), diabetes mellitus (OR = 0.027, 95% CI = 0.001–0.477), and JOA score (OR = 0.698, 95% CI = 0.526–0.927) were correlated with the occurrence of kinesiophobia after CSM surgery (*P* < 0.05). No significant multicollinearity was observed among the variables, with variance inflation factors ranging from 1.082 to 1.478.

**Table 3:** Multivariate Logistic regression analysis of kinesophobia in patients with CSM after operation

Variable	B	S.E.	Wald	*p*	Exp(B)	Lower 95%Cl for Exp(B)	Upper 95%Cl for Exp(B)
Age	0.100	0.044	5.151	0.023	1.105	1.014	1.204
Duration of symptoms	0.015	0.014	1.106	0.293	1.015	0.987	1.043
Diabetes mellitus (yes)	-3.636	1.478	6.055	0.014	0.026	0.001	0.477
Education levels (High school or below)	-0.195	0.892	0.048	0.827	0.823	0.143	4.725
JOA	-0.359	0.145	6.157	0.013	0.698	0.526	0.927
SAS	0.053	0.067	0.608	0.436	1.054	0.924	1.203
NDI	0.237	0.069	11.955	0.001	1.268	1.108	1.451
ADL	-0.066	0.047	1.978	0.160	0.936	0.853	1.026

JOA: Cervical Spine Japanese Orthopaedic Association; NDI: Neck Disability Index; SAS: Self-rating Anxiety Scale; ADL: Assessment of Activities of Daily Living and Functional State. Continuous variables are entered with original values, and categorical variables are assigned with values. High blood lipid: yes = 1, no = 0; Education: Junior high school or below = 1.Diabetes (yes) = 1;Diabetes (no) = 0

## Discussion

Our study revealed a high incidence of postoperative kinesiophobia in patients with CSM, at 75.4%. This raises significant concerns regarding rehabilitation outcomes. This high incidence may challenge the rehabilitation process, impacting patients’ exercise participation and rehabilitation effectiveness. The incidence rate found in our study is higher than rates reported by Bilgin et al. [19] for patients with chronic neck and waist pain (53.4%) and by Doury-Panchout et al. [20] for French patients (35.96%). This disparity may be attributed to perceived threats, follow-up time, sample size, regional differences, and cultural backgrounds.

Similar to Stubbs [21], we found that older age was a risk factor for kinesiophobia (*R* = 1.015). This may be due to the following reasons: this may stem in part from older patients’ more realistic concerns about the recovery process, including physical and psychological challenges. Regarding physiological mechanisms, as we age, Patients may experience changes such as decreased bone density and decreased muscle mass [22], which may lead to lower confidence in the sport. This physical discomfort may cause concerns about exercise, which in turn affects recovery after surgery. Regarding psychological mechanisms, there was a correlation between age and a patient’s understanding of the risks of surgery and the recovery process. Older patients may be more concerned about potential complications, which may exacerbate the fear of exercise by affecting their mental state. Therefore, rehabilitation needs to pay special attention to the patient’s cognitive level and provide targeted information and support to reduce their anxiety.

This study observed a significant association with diabetes mellitus in the development of postoperative kinesiophobia in patients with CSM. The association is likely the result of a complex mix of factors, providing a new perspective for understanding the formation mechanisms and management strategies of postoperative kinesiophobia. One explanation is that people with diabetes may be more focused on overall health, including the health of the skeletal and muscular systems [23]. This health awareness may make them more willing to actively participate in the rehabilitation movement and reduce worries about the rehabilitation process. In addition, People with diabetes often have greater self-regulation in managing chronic diseases, may to the rehabilitation process has a positive attitude, reduce the resistance of postoperative motion [24]. In addition, diabetes itself affects the nervous and motor system [25], which may provide earlier rehabilitation therapy, thereby reducing the occurrence of postoperative kinesiophobia. Therefore, in future studies, the physiological, psychological and behavioural characteristics of patients with diabetes need to be considered more comprehensively to gain a deeper understanding of their performance in postoperative kinesiophobia. Finally, the findings also suggest that there may be common genetic or environmental factors that influence the pathogenesis of cervical spondylosis and diabetes. This commonality may involve specific genotypes or environmental exposures that make it easier for a subset of patients with both diseases. Future studies are needed to further explore these potential mechanisms to better understand the relationship between postoperative CSM and diabetes, and to provide more precise targets for clinical intervention.

Despite the high incidence of postoperative kinesiophobia in patients with CSM, our study revealed significant improvements in functional outcomes. The correlation analysis results of postoperative kinesiophobia and functional rehabilitation outcomes of patients with CSM showed that postoperative TSK scores were significantly positively correlated with NDI, and negatively correlated with JOA scores three months after surgery. This showed that despite the patient’s kinesophobia, upper limb motor function, lower limb motor function, bladder function and cervical vertebra function were improved after decompression surgery. The fear-avoidance model explains the main cause of dysfunction in people with kinesiophobia, as fear may lead to avoidance of functional behaviours such as walking (stairs), neck movement, and bending over, which can lead to dysfunction and disability. The relationship of kinesiophobia with the cervical JOA score and NDI in this study was consistent with Guney-Deniz et al. [26]. , showing that kinesiophobia can affect the early prognosis, pain level, and joint function recovery after total knee arthroplasty. Jan J.M. Poo randomly grouped 146 patients with subacute neck pain and conducted follow-up at 12 and 52 weeks revealing that psychological factors had a significant impact on postoperative recovery, and exercise fear may hinder recovery in patients in the early or late postoperative period [27]. This finding highlights the close correlation between the patient’s psychological state and cervical spine function, especially in the early stages of recovery after surgery. Our results suggest that the postoperative rehabilitation program should not only focus on physical rehabilitation, but also intervene according to the psychological state of patients to improve their acceptance and participation in rehabilitation. The choice of a three-month follow-up period in our study was based on the need to capture early postoperative outcomes and to minimize potential confounders that may arise from longer follow-up periods. However, it is important to note that the impact of kinesiophobia on long-term functional recovery remains unclear. Future studies with longer follow-up periods are warranted to investigate the long-term effects of kinesiophobia on postoperative outcomes in CSM patients.

## Conclusion

This study delves into the ramifications of exercise fear on the postoperative rehabilitation outcomes of patients with Cervical Spondylotic Myelopathy (CSM). The findings reveal that a considerable 75.4% of CSM patients developed kinesiophobia post-surgery. Older patients exhibited a higher incidence of postoperative kinesiophobia, whereas patients with diabetes mellitus showed a lower incidence. This study demonstrates a prevalent occurrence of postoperative kinesiophobia in CSM patients. However, the three-month follow-up results indicate that despite the presence of kinesiophobia, patients showed improvement in upper and lower limb motor functions, bladder function, and neck function (NDI score). This finding suggests that although kinesiophobia may impact the rehabilitation process, patients still achieved significant recovery. Therefore, we recommend future rehabilitation programs to not only focus on physical recovery but also intervene based on the patients’ psychological state to enhance their acceptance and participation in rehabilitation.

## Limitations and future directions

Several limitations should be considered when interpreting the results of this study. First, the study had a relatively small sample size, which may limit the generalizability of the findings. Future studies with larger and more diverse samples are needed to confirm these results; Second, this study relied on self-report measures for assessing kinesiophobia, functional status, neck disability, and anxiety. Objective measures, such as physical performance tests or clinical assessments, could provide more accurate and reliable data; Third, the study was conducted at a single center, which may limit the generalizability of the findings to other populations or settings. Future multi-center studies are needed to validate these results in different populations; Finally, the cross-sectional design of the study limits the ability to draw causal conclusions. Longitudinal studies are needed to explore the temporal relationship between kinesiophobia and its associated factors over time.

In conclusion, this study explored the impact of exercise fear on postoperative rehabilitation outcomes in patients with CSM. The findings suggest that a high incidence of kinesiophobia after surgery may challenge the rehabilitation process, affecting patients’ level of exercise participation and rehabilitation effectiveness. Future research should focus on methods to alleviate patients’ fears, such as psychotherapy during recovery, biofeedback therapy, and encouraging a positive attitude towards exercise, to enhance physical performance and improve functional recovery after CSM surgery.

## Data Availability

The data supporting the findings of this study are available from the Department of Neurosurgery, Fujian Medical University, Union Hospital, but restrictions apply to the availability of these data, which were used under license for the current study, and so are not publicly available. However, data can be obtained by contacting the corresponding author if the author makes a reasonable request and obtains permission from the Department of Neurosurgery, Fujian Medical University, Union Hospital.

## Abbreviations

CSM: Cervicalspondylotic myelopathy
TSK: Tampa Scale of kinesiophobia
VAS: Visual Analogue Scale
SAS: Self-rating Anxiety Scale
JOA: Japanese Orthopaedic Association Score
NDI: Neck disability index
ADL: Activity of Daily Living
